# Comparing the influence of front-of-pack nutrition labels on Saudi consumers’ understanding and food selection

**DOI:** 10.3389/fpubh.2025.1527531

**Published:** 2025-06-03

**Authors:** Alaa Ashraf AlQurashi, Sumayyia D. Marar, Mohsen Ayyash, Halah Z. AlRawi, Amani Abu-Shaheen

**Affiliations:** ^1^Research Center, King Fahad Medical City, Riyadh Second Health Cluster, Riyadh, Saudi Arabia; ^2^School of Mathematical Sciences, Universiti Sains Malaysia, Penang, Malaysia

**Keywords:** nutrition labels, dietary habits, food, nutrition, calorie intake, obesity

## Abstract

**Introduction:**

Front-of-pack labels (FoPLs) are key public health tools that help consumers identify healthier food options. Although widely studied, little is known about their effectiveness in Saudi Arabia. This study aimed to determine the most understandable FoPL among five international systems to help Saudi consumers make healthier food choices.

**Methods:**

From January 1, 2022, to January 30, 2023, 2,509 Saudi consumers aged 18 years and above were recruited in public places across Riyadh. Participants were asked to select one product from sets of five food categories (bread, cheese, cereals, nuggets, and juice) with different nutritional profiles and then rank the products within each set based on their perceived nutritional quality. These tasks were first performed without any FoPL. Participants were then randomly assigned to one of the following five FoPL systems: Health Star Rating (HSR), Guideline Daily Amount (GDA), Multiple Traffic Lights (MTL), Chilean Warning Octagons (CWO), or Nutri-Score (NS), and asked to repeat the same tasks with the assigned label displayed on the packaging. Multivariate ordinal logistic regressions were performed to analyze whether changes in the scores of food choices and the ability to correctly rank the products were associated with the FoPL types, along with various socioeconomic and behavioral factors.

**Results:**

The analyses showed that participants improved their food choices depending on the FoPL format and the food category. Nutri-Score (NS) demonstrated a significant improvement in food choices across all food categories (OR = 1.96, 95% CI: 1.24 to 3.17, *p* = 0.003), particularly for nuggets (OR = 2.18, 95% CI: 1.16 to 3.17, *p* = 0.038) and cereals (OR = 2.16, 95% CI: 1.28 to 4.53, *p* = 0.001), compared to the GDA label. All FoPL types resulted in a greater proportion of correct responses in the ranking task compared to the no-label condition. Furthermore, NS emerged as the most influential FoPL in enhancing participants’ understanding of nutritional quality, significantly improving their ability to correctly rank products across all food categories (OR = 5.81, 95% CI: 2.92 to 7.28, *p* < 0.001).

**Conclusion:**

The study suggested that the presence of FoPLs can enhance consumers’ ability to evaluate the nutritional quality of food products. In particular, the study demonstrated that NS is among the most effective FoPLs that help Saudi consumers assess the nutritional quality of different food categories. Policymakers may consider adopting NS as a standard FoPL system to support healthier dietary behaviors and reduce the prevalence of diet-related diseases in Saudi Arabia.

## 1 Introduction

Obesity and unhealthy dietary habits remain a public health issue worldwide, with high prevalence, as approximately 890 million adults are obese (1, 2). Eating habits have changed worldwide; many people follow an unhealthy diet with poor nutrition through pre-packed and processed food, which generally contains high levels of sugar, fat, saturated fatty acids, trans-fatty acids, and sodium (3). This low quality of food selection is related not only to obesity but also to several chronic diseases such as heart disease, stroke, and diabetes (4).

In Saudi Arabia, recent research has shown that the prevalence of obesity among the adult population has been a cause for concern, fluctuating between 22% in 2020 and 21.4% in 2023 (5). Furthermore, a cross-sectional study reported that the weighted prevalence of obesity among Saudi adults was 24.7% in 2020, indicating a pressing need for effective interventions (6). A representative national survey on nutrient and energy intake demonstrated that almost 80% of adults exceed the daily fat intake recommendations (30% of total energy intake) (7). Childhood obesity is also considered high in Saudi Arabia (8). A study by Al Shehri et al. (3) reported that childhood obesity was associated with higher intakes of fast food and other factors.

Although genetic factors are linked to developing a chronic disease, controlling modifiable factors could reverse the condition based on the latest evidence. For example, modifying lifestyle and nutrition significantly impacts prevention (9). In this context, the World Health Organization (WHO) recommends monitoring the consumption of poor nutrients (10). However, the growing prevalence of processed and packaged foods has made it challenging for consumers to evaluate the nutritional quality of their purchased products. Nutrition labels are often too complicated for the average consumer to interpret, and the widespread use of health and nutrition claims further confuses them (11, 12).

As a public health strategy to control the rapid growth of obesity and its comorbidities in the population and the health system, policymakers worldwide implemented front-of-pack nutrition labelling (FoPL). This method aimed to promote healthier eating habits by providing consumers with clear and easily understandable nutrition information, enabling them to make better food choices at the point of purchase (13–15). Various interpretive FoPL systems have been implemented globally to assist consumers in making informed dietary choices (16). Notable examples include the Multiple Traffic Lights system in the United Kingdom and Ecuador, the Nutri-Score (NS) adopted in several European countries (i.e., France, Belgium, Germany, Spain, the Netherlands, Luxembourg, and Switzerland), and Warning Labels (WL) introduced in Chile, Mexico, Uruguay, and Peru. Additionally, the Health Star Rating system (HRS) is utilized in Australia and New Zealand. Another category of interpretive FoPLs consists of endorsement logos, such as the Nordic Keyhole (Northern Europe), the Choices Programme (Czech Republic, Nigeria), and the Healthier Choice Symbol (Singapore) (17, 18). The scoring system in FoPL models assesses key nutrients to simplify nutrition information, guiding consumers toward healthier choices. It also encourages manufacturers to improve product formulations, contributing to better public health outcomes by promoting a healthier food environment and reducing diet-related diseases (19, 20). In Saudi Arabia, the Saudi Food and Drug Authority (SFDA) introduced mandatory menu calorie labelling regulations in January 2019, requiring restaurants and cafes to display comprehensive nutritional information beyond just calorie content, including proteins, fats, carbohydrates, and sugars (21). This initiative aims to improve nutritional consumption among Saudis to control the low quality of food, provide appropriate nutrition facts for the consumers, and also monitor freshness and hygiene standards of packed-cooked food (22).

In recent years, bibliometric analyses have highlighted the increasing research focus on FoPL systems, particularly since 2018, indicating a growing academic interest in assessing their effectiveness. The USA leads in scientific production, followed by Brazil and Chile (23). FoPL is proven internationally to be a cost-effective strategy to encourage healthier eating habits and reduce the risk for several nutrition-related diseases by giving consumers clear, easy-to-understand nutrition information (14, 24). Several theories have been explored in relation to the effectiveness of various types of food labels. For instance, the dual-process theory was used to examine consumers’ responses to different FoPL formats (25), while another study used the Health Belief Model to assess the frequency of using and intentions to use red/green FoP labels in the future (26). The Guideline Daily Amount (GDA) labels are the more common design, which shows the amount of fat, saturated fat, sugar, and sodium in grams as well as the kilocalories per portion and the percentage (27). The Multiple traffic lights (MTL) label is used in the UK and has the corresponding color for the content of fat, saturated fat, sugar, and sodium. Green, amber, and red represent low, moderate, and high contents of the nutrients in the food product, respectively. The colors are based on concentration in grams per 100 g or per 100 mL, and the criteria of the UK Food Standards Agency were applied to assign the color codes (28). Also, calories were shown in a neutral color (white/grey). Moreover, Chilean Warning Octagons (CWO) are another valid tool that provides clear and comprehensive information to the consumer on nutrients that, when consumed in excess, can cause health problems. CWO applies to all national/imported packaged foods and beverages with added sodium, sugars, or saturated fat (29). The Nutri-Score (NS), implemented in France in 2017 and later in 2018 in Belgium and Spain, characterizes the overall nutritional quality of the food or beverage using a graded scale of five colors from dark green (associated with the letter A) to dark orange (associated with the letter E).

The findings of several studies indicated that different FoPL types enhance consumers’ awareness and understanding of food product healthiness (28, 30, 31). However, interpretive FoPLs (e.g., NS, Warning Labels) are more effective than reductive models (e.g., GDA) in guiding consumers toward healthier food choices (18, 28, 32–39). For example, evidence from international comparative studies demonstrated that the NS was the most effective label, followed by the MTL, HSR, WL, and Reference Intakes (RI) across different types of food products (18, 36, 38). In Saudi Arabia, a randomized control trial showed that both NS and WL were effective in improving the purchasing behavior of food and beverages, but NS showed to be the effective model when the consumer targeted diet quality (34). However, there is limited evidence comparing the impact of different international FoPL designs on eating behavior across various societies, particularly in Saudi Arabia. Previous research also indicated that the Saudi population had low to moderate awareness and knowledge about nutrition and food labels (40–43). Despite global efforts, Saudi Arabia lacks a standardized FoPL system. The absence of a specific FoPL graphic design in Saudi Arabia limits consumers’ ability to make informed dietary choices. Additionally, there is a lack of studies comparing the effectiveness of various FoPL formats among Saudis.

To overcome this gap, the current study takes a step further by investigating the impact of five different FoPL systems on Saudi consumers’ understanding and food selection. More specifically, the study aims to identify the easiest and most understandable internationally used FoPL graphic design for Saudi consumers by assessing their comprehension of the five FoPLs and examining the association between different FoPL graphic designs and consumers’ food choices.

## 2 Materials and methods

### 2.1 Study design

An experimental study with five different labels was carried out between 1 January 2022 and 30 January 2023. The five specific FoPLs included in this study were selected based on their relevance and widespread use in international regulations, demonstrated impact on consumer understanding, and relevance to the Saudi market. These labels represent a mix of interpretive and nutrient-specific formats, allowing for a comprehensive evaluation of their influence on consumer choices. Furthermore, previous research has identified these selected FoPLs as among the most commonly implemented or recommended worldwide, making them particularly suitable for comparison (18, 36, 37).

Building on the methodology of Egnell et al. (36), this study was adapted to the Saudi context by implementing Arabic language translation to ensure clarity and comprehension, selecting food products commonly consumed in Saudi Arabia to enhance relevance, and incorporating a diverse consumer sample that reflects variations in educational background, nutritional awareness, and purchasing behavior. Moreover, modifications were made to the presentation and formats of FoPLs to align with local reading habits and cognitive processing preferences, ensuring that the study accurately captures how Saudi consumers interpret and respond to different labelling systems. Therefore, the study explicitly tested the hypothesis that different FoPL formats influence Saudi consumers’ ability to understand nutritional information and make healthier food choices to varying degrees.

### 2.2 Study population

Both males and females, 18 years old and above, were approached at public places in Riyadh, Saudi Arabia, to take part in this study, considering different socioeconomic statuses. People who refused to participate or who did not purchase the food products proposed in this study were excluded. The study focused on individuals who purchase food products to ensure the findings directly apply to real-world decision-making processes. Since FoPLs are intended to guide purchasing behavior, evaluating their effectiveness among those actively engaged in food selection is crucial. Including non-purchasers could introduce variability unrelated to actual consumer decision-making, potentially diluting the study’s primary objective of assessing how FoPLs influence purchasing choices. However, it is acknowledged that non-purchasers may still possess valuable insights regarding label interpretation.

### 2.3 Sample size calculation

The sample size was determined in two stages. In the first stage, the minimum required sample size for each arm was established through power analysis using PASS software, version 11. Establishing a 95% confidence interval with a power of 80%, and an expected proportion of 0.44 for correctly identifying FoPLs based on a pilot test and previously published studies (17). Consequently, the minimum required sample size for each arm was determined to be 484 participants. Therefore, the study needed to recruit at least 2,420 participants across the five arms, assuming equal allocation per arm. In the second stage, stratification was performed using quotas based on sex (1:1) and age groups. The distribution for age groups was as follows: 18 to 29 years (33.0%), 30 to 39 years (33.0%), 40 to 49 years (20.0%), and 50 years and above (16.0%). These percentages were determined according to the General Authority for Statistics in Saudi Arabia for Riyadh City.

### 2.4 Survey development

The questionnaire was developed in the Arabic language based on previously published studies (24, 36, 37). A group of ten nutritionists and clinical researchers from different countries with expertise in research and publication, reviewed the questionnaire and provided feedback on its face and content validity. The panel further validated the questionnaire across all five study arms using the Scale-Content Validity Index/Average (S-CVI/Ave), with a score of 0.8 or higher indicating agreement (calculation details are not shown). Following this validation, a pilot study with 50 participants was conducted to assess readability, comprehension, clarity, and language accuracy. The questionnaire was then modified based on the feedback from the participants. The study questionnaire includes demographic, lifestyle, and nutrition-related questions. Participants were asked to report the frequency with which they purchase items from specific food categories to verify that their responses accurately reflect actual food choice behaviors. Participants who answer “Never” to at least two out of the three food categories will be excluded from the study. Additionally, the study includes a food choice task and a ranking task (to assess objective understanding), with the scenarios for ranking and selection explained earlier.

Moreover, the questionnaire included questions regarding the factors people consider when reading FoPL, including: (1) For what bibliometric do you never or rarely use nutritional labels on the packaging of packaged foods and bottled beverages? (2) Why would you sometimes or almost always use the nutritional labels on packaged foods and bottled beverages? The responses to questions 1 and 2 were through multiple options that allowed participants to check the statement that most closely matched their beliefs. Question 3 was: Do you know what harmful content can be identified from reading the nutrition information on the front labelling? Responses to this question included commonly reported ingredients such as saturated fat, high sodium, protein, carbs, fiber, vitamins and minerals, high sugar, preservatives, and the option “I do not know.”

### 2.5 FoPL’s design and stimuli

The food categories chosen for stimulus development were selected based on the significant variation in nutritional quality within each category and their widespread consumption in Saudi Arabia. To avoid potential biases arising from factors such as brand recognition, loyalty, or established habits, fictional product packaging featuring the invented brand name “Stofer” was employed as the stimulus material.

The simulated packages were designed to closely resemble actual food products, with a zoom feature included to allow participants to magnify specific sections, such as the FoPL. Within each food category, three products were created with different nutritional profiles (i.e., low, medium, and high quality) to enable ranking. These products were consistent across all FoPL variations. To avoid influencing participants’ perceptions, no additional nutritional details or quality markers (e.g., organic certification) were displayed on the simulated packages. All FoPL variants were placed in the same location on each package and covered a similar surface area.

This study tested five FoPLs, including both nutrient-specific and summary systems. The nutrient-specific labels were: (1) the MTL^1^, which uses color coding (green, amber, red) to indicate low, medium, or high levels of key nutrients (i.e., energy, fat, saturated fat, sugar, and salt), respectively; (2) the GDA^2^, a one-color label showing numerical values for key nutrients; and (3) the CWO^3^, which flags products that exceed recommended levels of certain critical nutrients. The summary labels included: (4) the NS^4^, a five-color ordinal scale from dark green (A) to dark orange (E) representing overall nutritional quality; and (5) the HSR^5^, which rates products from half a star to five stars based on their overall healthiness.

**Figure 1:**
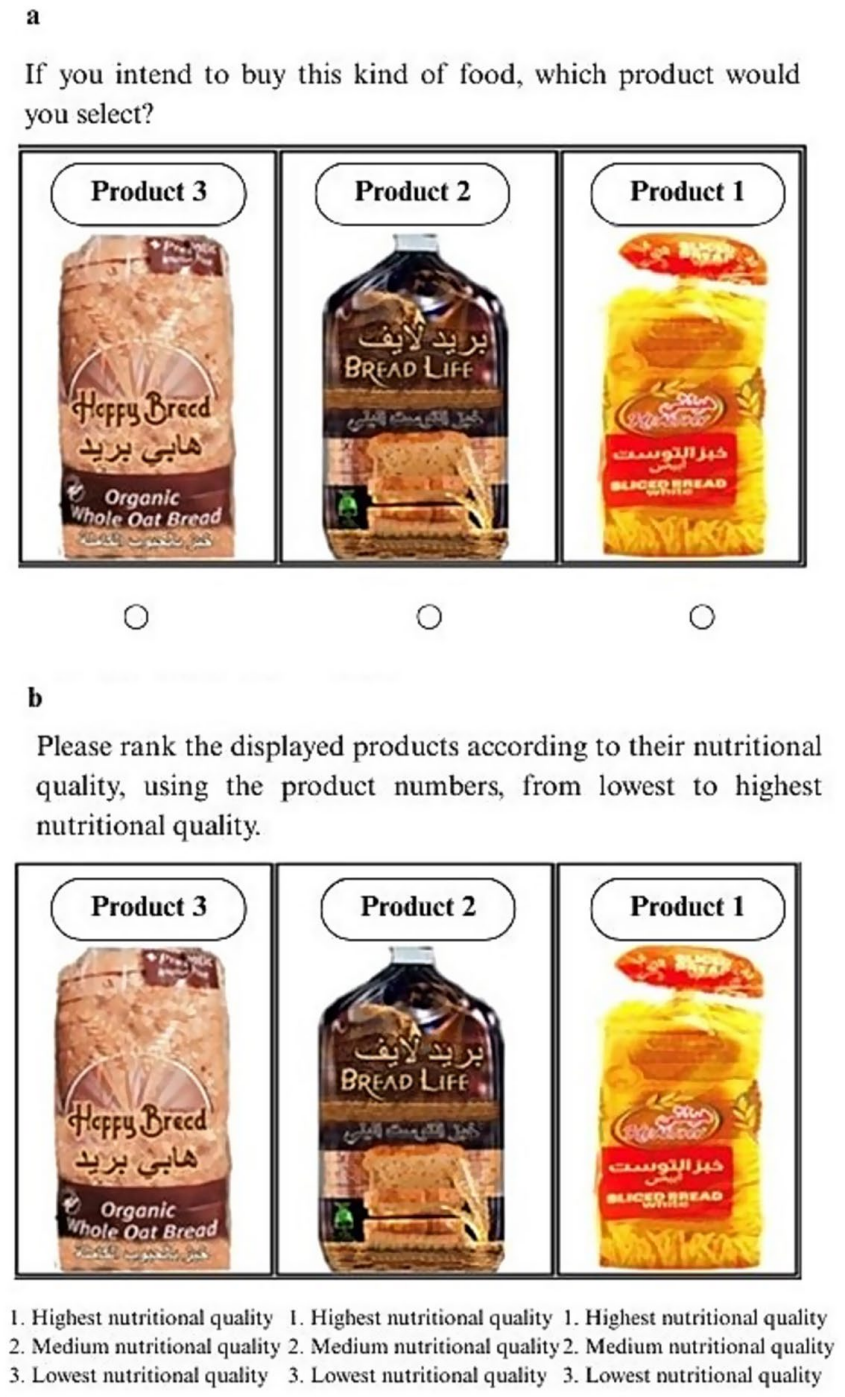
Procedure of the choice and ranking tasks for the bread product category without FoPL. a: Food choice task without FoPL. b: Ranking task without FoPL.

### 2.6 Data collection procedure

The research team recruited data collectors to collect data from public areas in Riyadh City during the study period. They were trained on how to approach respondents and collect data. The data collectors provided participants with an online survey, which included the study questionnaire described in the survey development section.

The procedure for data collection involved two stages. In the first stage, participants were exposed to five types of food items, with each set containing three images representing a specific food category, all without any labels on the front of the simulated packages (referred to as the “No Label Phase”). Based on the dietitian’s recommendation, the selected food categories included dairy products (cheese), beverages (juice), ready-made products (nuggets), and grain products (cereals and bread). To minimize potential bias in product evaluation, the simulated packages featured an imaginary brand. For each food category, participants were asked to complete two tasks: selecting a product they would buy (“Food Choice Task”) and ranking the products based on their perceived nutritional quality (“Ranking Task”). In the food choice task, participants selected which of the three displayed products they would purchase, with an option to choose “I would not buy any of these products” also available. Figure 1a illustrates the food choice task without FoPL for the bread product as an example. Following each choice task, participants completed the ranking task, where they were asked to rank the three products based on their nutritional quality: 1 for “Highest nutritional quality,” 2 for “Medium nutritional quality,” and 3 for “Lowest nutritional quality.” An “I do not know” option was also available, as shown in Figure 1b.

**Figure 2:**
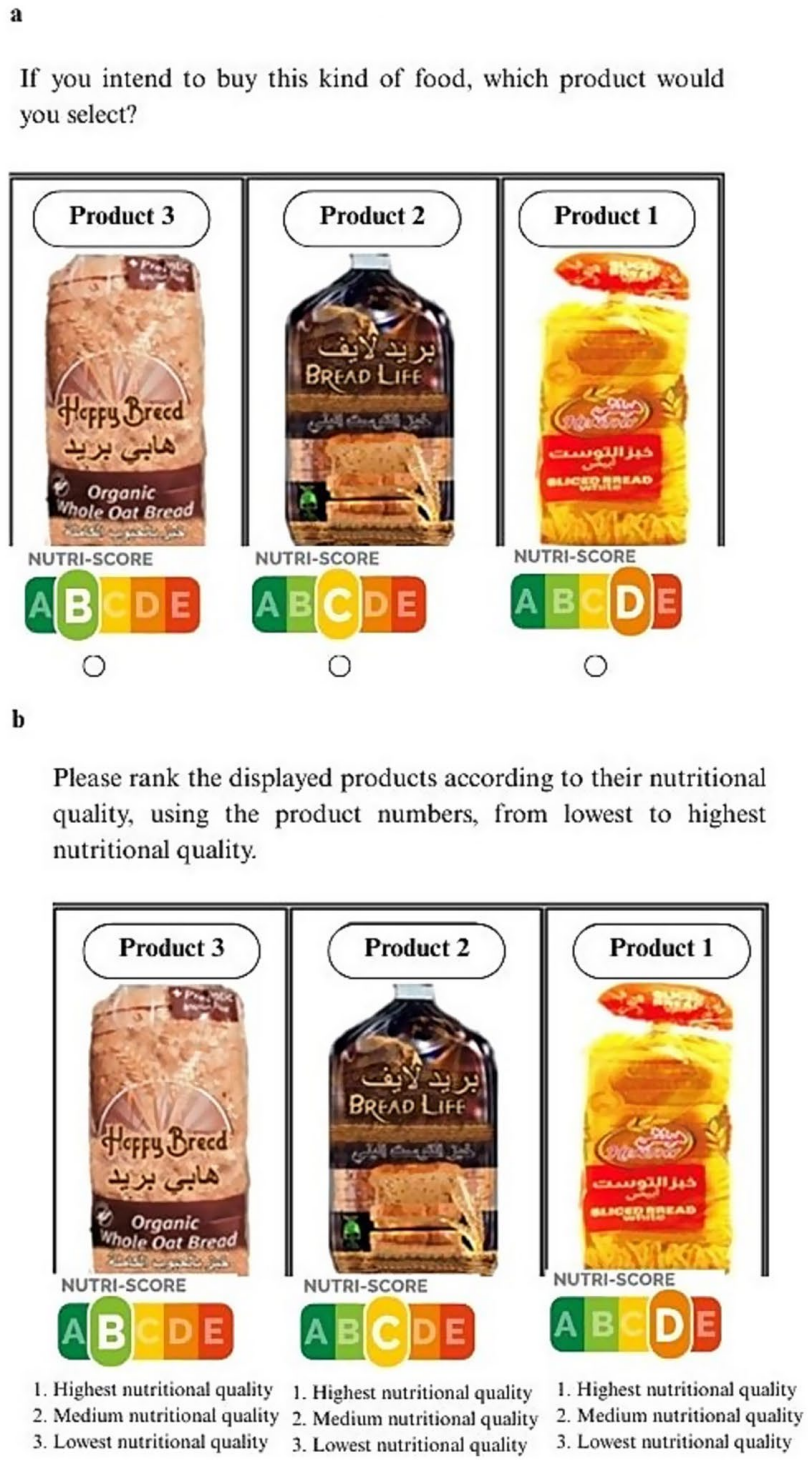
Procedure of the choice and ranking tasks for the bread product category with FoPL. a: Food choice task with FoPL. b: Ranking task with FoPL.

In the second stage, participants were randomly assigned to one of five FoPL groups: Group A (GDA), Group B (MTL), Group C (HSR), Group D (CWO), and Group E (NS). These mock labels were applied to the same food products. The randomization list was generated using a computer-based system. To ensure equal distribution across demographic groups, stratified randomization was applied using predefined quotas for gender and age, maintaining balanced representation within the sample. Participants were then asked to complete the same two tasks conducted in the first stage (food choice and ranking tasks), this time with the FoPLs visible on all products (the “FoPL Phase”). Figures 2a,b illustrate the food choice and ranking tasks, respectively, conducted with the presence of FoPLs for the bread category. The FoPLs were consistently placed in the same location on each food product. To prevent undue influence on participants’ perceptions, no additional nutritional information or quality indicators were provided. Moreover, participants were not informed that they would be shown the same products again or that labels would be added.

### 2.7 Data analysis

#### 2.7.1 Descriptive statistics

Demographic, lifestyle, and nutritional characteristics were described using means and standard deviations for quantitative continuous variables, while counts and percentages were used to describe categorical variables. For calculating participants’ knowledge scores, correct answers (yes) were assigned a score of one, while incorrect answers (no/not sure) or unanswered questions were assigned a score of zero.

#### 2.7.2 Food choices

In evaluating choices, participants were assigned a numerical score based on their selections. They received a score of +1 for choosing the product with the lowest nutritional quality, +2 for selecting the product with medium nutritional quality, and +3 for opting for the product with the highest nutritional quality. Based on previously published studies, this scoring system was initially implemented without any labelling and later applied when FoPLs were present (18, 36, 37). Consequently, for each food category, we computed a score by taking the difference between the points allocated in one condition and those in the other. This yielded a discrete, continuous score ranging from-2 to +2 points. To establish an overall score, we then summed the scores from each category, producing a final score that could range from-6 to +6 points. The calculation process involved evaluating the proportion of participants within each FoPL group who showed a shift, whether a deterioration or an improvement, in their food choices when transitioning from the condition without labels to the condition with FoPLs. This assessment was performed independently for each specific food category. For each food category, the proportions of respondents whose food choices improved or deteriorated between the No Label and FoPL phases were computed for each FoPL group, and the findings were visually represented using clustered bar charts.

Spearman’s correlation coefficients were initially used to examine the associations between food choice scores, types of FoPLs, and sociodemographic and behavioral variables across all food categories. Multivariable ordinal logistic models were executed to assess the association between choice score and FoPL type (HSR, MTL, NS, and CWO, with GDA as the reference) after adjustment for socio-demographic variables and behavioral variables that have prior empirical and theoretical significance (37–39, 44, 45). Demographic variables such as age, sex (female as the reference category), and marital status (single as the reference category) were included. Furthermore, education level and income level were treated as ordinal variables to account for the inherent order of categories. Behavioral variables included responsibility for grocery shopping, self-assessed diet quality, nutrition knowledge, and awareness of the label during survey completion. For binary variables, the reference category was ‘No’, and for diet quality, ‘Unhealthy’ served as the reference category. The models were estimated for the overall sample, irrespective of food category, as well as separately for each food category. To address potential multicollinearity, variance inflation factors (VIFs) were calculated for each predictor variable. Variables with VIF exceeding a threshold of 10 were considered highly collinear and were either excluded or combined with other related variables to mitigate multicollinearity. The analysis included only respondents who had chosen a product in both the no-label and FoPL conditions. Statistical significance was determined at a *p*-value threshold of less than 0.05.

#### 2.7.3 Objective understanding

This study assessed consumers’ objective understanding of the FoPLs by analyzing their ability to accurately rank groups of products based on their nutritional quality. A response was considered accurate when all three products in a set were correctly ranked, leading to the assignment of +1 point for that particular category. Participants who made one or more mistakes in the ranking task were given a score of-1 point. However, if participants chose the “I do not know” option, no points were assigned. This scoring system, based on previously published studies, allowed for the calculation of a ranking ability score for each food category by subtracting the points obtained in the no-label condition from those in the FoPL condition (18, 36, 37). This resulted in a score range of-2 to +2 points for each category. Subsequently, a global score, encompassing all three food categories, was determined, with a potential range from-6 to +6 points. The calculation process involved assessing the percentage of accurate responses in both the absence of labeling and FoPL conditions, categorized by the type of FoPL and the particular food category, with the results visually presented using clustered bar charts.

The relationships between objective understanding scores, types of FoPLs, and various sociodemographic and behavioral variables were initially examined using Spearman’s correlation coefficients, across all food categories. The association between the objective understanding score and FoPL type was further examined by employing ordinal logistic regression models for the overall sample as well as for each food category, controlling for age, sex, education, income level, responsibility for grocery shopping, self-assessed diet quality, nutrition knowledge, and awareness of the label during survey completion, as defined in the previous section. In both ordinal logistic regression models, the GDA label served as the reference category for the FoPL categorical variable. All statistical analyses were performed using the Stata version 15 software package (StataCorp LP, College Station, TX).

### 2.8 Ethical consideration

Ethical approval has been obtained from the Institutional Review Board at King Fahad Medical City with KFMC IRB log # 20–033. The study participants were recruited voluntarily, and after they had read, understood, and signed the study informed consent.

## 3 Results

### 3.1 Baseline characteristics

Table 1 shows the characteristics of the study sample, including sociodemographic, lifestyle factors, and nutrition-related details. The sample consisted of 2,509 participants from Riyadh, Saudi Arabia, and its distribution according to sociodemographic characteristics revealed that 50.2% (*n* = 1,256) were men, 33.1% (*n* = 830) were aged 18 to 29 years, with an average age of 29.4 ± 10.3 years. Additionally, 64.3% (*n* = 1,614) had a university degree, 58.5% (*n* = 1,467) were single, 41.8% (*n* = 1,049) were employed, and 42.1% (*n* = 1,056) reported a medium monthly income level. The average household size was 6.1 ± 3.6 individuals, and the mean weight and height were 69.7 ± 18.7 kg and 163.8 ± 9.7 cm, respectively. Furthermore, more than half of the respondents (58.7%, *n* = 1,473) stated that they were responsible for grocery shopping. Moreover, 39.3% (n = 986) of participants reported that they mostly eat an unhealthy diet, and 12.5% (*n* = 314) stated that they eat a very unhealthy diet. Approximately half of the respondents (51.0%, *n* = 1,280) declared they had tried reducing their weight. During the survey, 25.9% (*n* = 649) of participants stated that they could not recall encountering the food label, with the highest percentage of such cases occurring among those assigned to the GDA label group.

**Table 1:** Descriptive statistics of the study sample (*n* = 2,509).

Categorical variables	*N*	%
Sex		
Male	1,259	50.2
Female	1,1,250	49.8
Age group		
18–29	830	33.1
30–39	781	31.1
40–49	485	19.3
≥ 50	413	16.5
Educational level		
Lower secondary	79	3.1
Secondary	503	20.0
University	1,614	64.3
Graduate	313	12.5
Marital status		
Single	1,467	58.5
Married	850	33.9
Widowed	43	1.7
Divorced	149	5.9
Employment status		
Employed	1,049	41.8
Unemployed	703	28.0
Student	757	30.2
Level of monthly income		
Low (< 10,000 SAR)	971	38.7
Medium (10,000–20,000 SAR)	1,056	42.1
High (> 20,000 SAR)	482	19.2
Responsible for shopping		
Yes	1,473	58.7
No	1,036	41.3
Self-rating diet quality		
Unhealthy diet	314	12.5
Most of my diet is unhealthy	986	39.3
Most of my diet is healthy	1,049	41.8
Healthy diet	160	6.4
Tried to reduce weight		
Yes	1,280	51.0
No	1,229	49.0
Did you see the FoPL label during the survey?		
Yes	1,540	61.4
No	649	25.9
Not sure	320	12.8
Respondents recall seeing the FoPL to which they were exposed		
HSR	268	55.4
MTLs	418	68.5
Nutri-Score	325	75.9
CWO	324	59.7
GDA	205	46.2

Continuous variables	Mean	SD
Age, years	29.4	10.3
Household size	6.1	3.6
Weight, kg	69.7	18.7
Height, cm	163.8	9.7

FoPL, Front-of-Pack Labels, SAR, Saudi Riyals; SD, Standard Deviation,

### 3.2 Knowledge and habits related to food labeling

Table 2 shows participants’ knowledge and habits related to food labeling. The results indicated that 21.6% (*n* = 541) of participants reported that they did not have nutrition knowledge, and 13.8% (*n* = 346) stated that they did not know food and beverage labels. Moreover, 21.3% (*n* = 535) of participants stated that they did not know the importance of food labels, and 19.2% (*n* = 481) were unaware of what information could be obtained from such labels. More than one-third of respondents did not know the amount of calories required for the human body per day (34.4%, *n* = 863) and did not know which ingredients should be checked when buying a product (37.1%, n = 930). Furthermore, 40.5% (*n* = 1,015) of participants reported that the quality of the product influenced their purchasing decisions. When asked about the frequency of reading food labels, 45.7% (*n* = 1,147) reported that they sometimes read them. Among those who reported reading food labels, 38.8% (*n* = 852) stated that they read them to know the harmful ingredients. The majority also indicated that they usually check for fat, fiber, protein, sodium, and/or carbohydrate percentages, vitamins and minerals, and/or industrial preservatives (77.2%, *n* = 1,695). The most common reasons for not reading food labels included lack of time, not knowing their uses, lack of interest, difficulty understanding them, and/or the availability of food labels in English (41.7%, *n* = 131).

**Table 2:** Knowledge and habits related to food labeling.

Item	*N*	%
Nutrition knowledge		
Yes	1,747	69.6
No	541	21.6
Not sure	221	8.8
Knowledge of food and beverages have nutritional labels		
Yes	1,894	75.5
No	346	13.8
Not sure	269	10.7
Do you know why these labels are important?		
Yes	1,632	65.0
No	535	21.3
Not sure	342	13.6
Do you know what information you can get from these food labels?		
Yes	1,604	63.9
No	481	19.2
Not sure	424	16.9
Knowledge of calories that the body needs per day		
Yes	1,260	50.2
No	863	34.4
Not sure	386	15.4
Knowledge of what ingredients must be checked for their presence in food products		
Yes	1,049	41.8
No	930	37.1
Not sure	530	21.1
Influences on the decision to purchase food products		
Product price	502	20.0
Product Quality	1,015	40.5
Both product price and quality	992	39.5
Reading food labels		
Always	477	19.5
Sometimes	1,147	45.7
Rarely	571	22.8
Never	314	12.5
Reasons for reading food labels (*n* = 2,195)		
To know the harmful ingredients	852	38.8
Need to know that it is necessary to know the ingredients used in the product	556	25.3
Follow a diet program to lose weight	98	4.5
At least two of the above reasons	689	31.4
Ingredients checking (*n* = 2,195)		
Sugar, fat, fiber, protein, sodium, and/or carbohydrate percentage	247	11.3
Vitamins and minerals	186	8.4
Industrial preservatives	67	3.1
At least two of the above ingredients	1,695	77.2
Reasons for not reading food labels (*n* = 314)		
Do not have time to read it	26	8.3
Do not know its uses	65	20.7
Not interested	21	6.7
Cannot understand it	35	11.1
Available in the English language only	36	11.5
At least two of the above reasons	131	41.7

### 3.3 Results of food choice analysis

**Figure 3:**
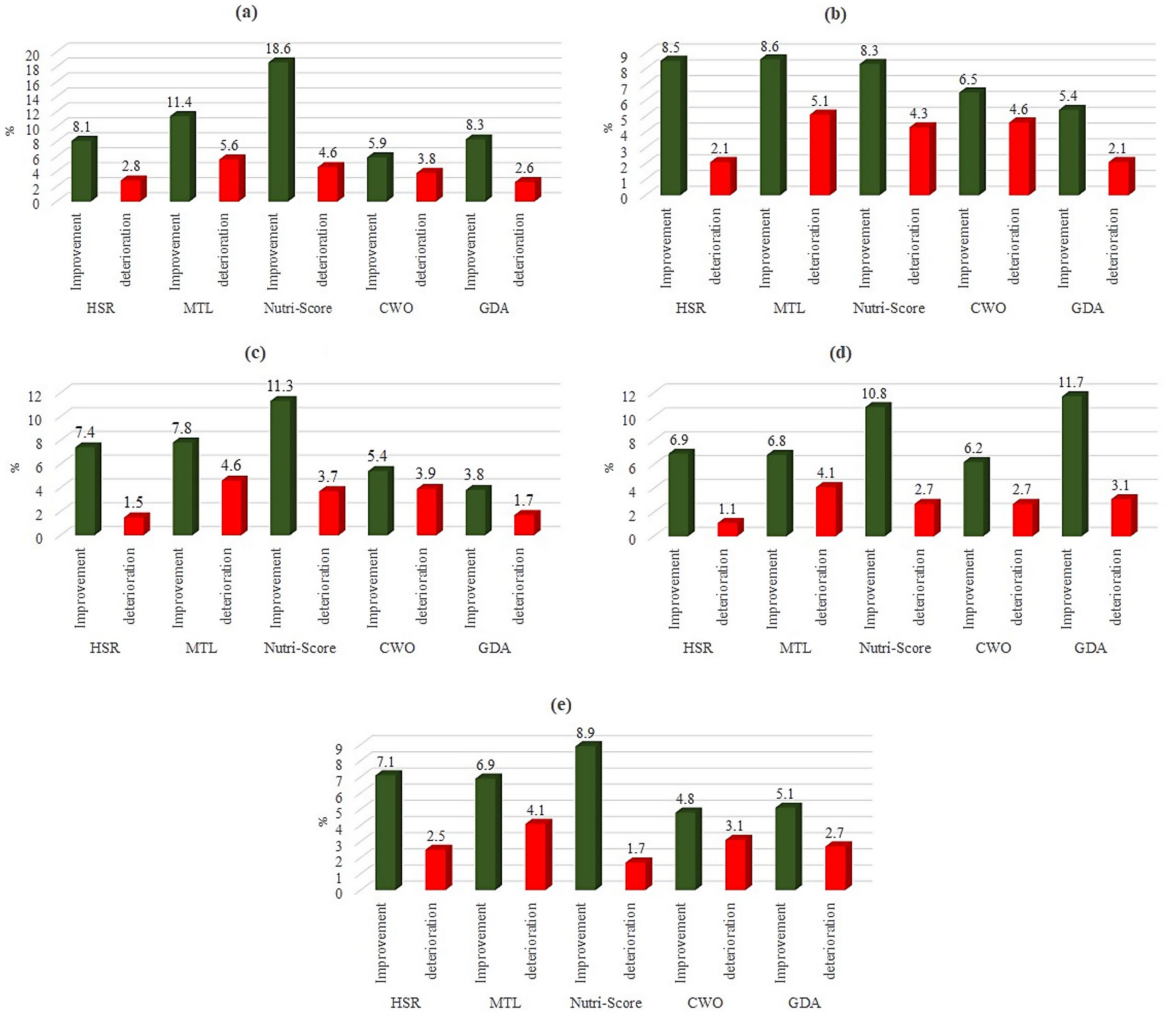
Proportions of improvement and deterioration of the nutritional quality of food choices, by FoPL and food types. (a): Cheese. (b): Nuggets. (c): Juice. (d): Cereals. (e): Bread.

A substantial number of participants either maintained consistent food choices between the two labeling situations, with percentages ranging from 62.4 to 74.9%, depending on the label and food category, or did not make any selections in one or both labeling conditions, with percentages ranging from 25.2 to 33.8%. Figure 3 indicates that, across all five food categories and five distinct FoPLs, a greater percentage of participants improved their food choices between the two labeling conditions compared to those whose choices deteriorated. However, these outcomes varied depending on the specific FoPL used, highlighting discrepancies in performance across different food categories. Overall, Figure 3 suggested that the NS label had the best performing FoPL at enhancing participants’ choices across most product types, with the highest rates of improvement for cheese, juice, and bread. However, its performance varied slightly by food category, as other FoPLs outperformed it in certain cases, such as GDA for cereals and MTL for nuggets. This variation suggests that, while NS generally enhances consumers’ ability to identify healthier options, the relative effectiveness of FoPLs may depend on the specific food category under consideration.

The preliminary correlation analyses in Supplementary Table S1 indicated that NS and the HSR were significantly and positively associated with healthier food choices, both in the overall sample and across most food categories. Moreover, older adults, higher education and income levels, better self-assessed diet quality, greater nutrition knowledge, and label awareness at the time of survey completion were positively correlated with healthier food choices across the entire sample and most food categories. In contrast, sex, marital status, and responsibility for grocery shopping were not significantly associated with food choice behavior.

Table 3 displays the multivariate ordinal logistic regression results for the change in nutritional food choices scores across all food categories combined and for each food category. All models performed well and passed all the diagnostic tests. The results indicated that among the different FoPL types, the NS was the only one that had a significant effect in improving the nutritional quality of food choices compared to the GDA label, after controlling for individual-level characteristics (OR = 1.96, 95% CI: 1.24 to 3.17, *p*-value = 0.003). The odds ratio of 1.96 suggests that participants exposed to the NS label were 1.96 times more likely to choose healthier food options than those who saw the GDA label, highlighting that the NS was nearly twice as effective as GDA in guiding consumers toward making better nutritional choices. The statistically significant result further supports the reliability of this finding, indicating that the NS may be a more effective front-of-pack label for encouraging healthier food selections. Specifically, among cheese products (OR = 2.18, 95% CI: 1.16 to 3.17, *p*-value = 0.038) and cereals (OR = 2.16, 95% CI: 1.28 to 4.53, *p*-value = 0.001), participants who were exposed to the NS label were more than twice as likely to make healthier choices compared to those who saw the GDA label. These findings suggest that the NS was particularly effective in influencing food choices within these categories, reinforcing its potential as a tool for promoting better nutritional decisions. However, no significant associations were found between food choices and the MTL, HSR, or CWO FoPLs when compared to the GDA label.

**Table 3:** Multivariate ordinal logistic regression results for the factors associated with the scores of food choices across food categories.

Variables	All food types		Cheese		Nuggets		Juice		Cereals		Bread	
	OR [95% C.I]	*p*-value	OR [95% C.I]	*p*-value	OR [95% C.I]	*p*-value	OR [95% C.I]	*p*-value	OR [95% C.I]	*p*-value	OR [95% C.I]	*p*-value
Type of FoPL												
HSR	1.25[0.82–2.24]	0.224	1.42 [0.62–3.15]	0.327	1.14 [0.46–2.15]	0.428	1.36 [0.63–2.57]	0.328	1.44 [0.68–2.37]	0.426	1.54 [0.79–3.12]	0.317
MTL	1.38[0.92–1.74]	0.637	1.21 [0.48–2.08]	0.826	1.08 [0.16–1.69]	0.724	1.24 [0.58–2.19]	0.524	1.78 [0.96–3.34]	0.087	0.86 [0.37–1.88]	0.931
NS	1.96* [1.24–3.17]	0.003	2.18* [1.16–4.29]	0.038	1.82 [0.86–3.15]	0.084	1.65 [0.89–3.24]	0.148	2.16* [1.28–4.53]	0.001	1.41 [0.76–2.62]	0.247
CWO	0.94 [0.42–1.36]	0.762	1.03 [0.26–2.48]	0.925	1.38 [0.72–2.73]	0.486	1.29 [0.72–2.63]	0.526	1.41 [0.76–2.62]	0.247	0.87 [0.41–1.79]	0.492
Male	0.97 [0.85–1.23]	0.836	0.92 [0.66–1.42]	0.762	1.02 [0.75–1.38]	0.891	1.10 [0.83–1.46]	0.498	1.06 [0.76–1.92]	0.671	1.36 [0.67–2.73]	0.427
Marital status												
Married	1.07 [0.88–1.62]	0.511	1.12 [0.78–1.94]	0.532	0.95 [0.69–1.31]	0.755	1.02 [0.76–1.88]	0.897	1.13 [0.58–2.82]	0.426	1.12 [0.83–2.52]	0.447
Divorced	0.98 [0.65–1.46]	0.924	0.91 [0.51–1.63]	0.788	0.89 [0.51–1.58]	0.695	0.95 [0.55–1.66]	0.855	1.15 [0.61–2.18]	0.660	1.12 [0.79–2.14]	0.627
Widowed	0.90 [0.56–1.45]	0.672	1.02 [0.48–2.18]	0.953	1.12 [0.54–2.34]	0.762	1.21 [0.61–1.74]	0.579	1.22 [0.64–2.48]	0.642	1.24 [0.64–2.27]	0.565
Age	1.95* [1.41–2.86]	0.002	1.74* [1.29–2.33]	0.008	1.05 [0.81–1.37]	0.721	1.18 [0.85–2.93]	0.136	1.46* [1.11–2.86]	0.019	1.59* [1.19–2.13]	0.016
Education	2.48* [1.27–4.98]	0.006	1.71* [1.12–3.21]	0.031	2.66* [1.58–6.15]	<0.001	3.41* [1.41–6.24]	<0.001	2.46* [1.27–4.77]	0.001	3.28* [2.21–4.96]	0.000
Income level	3.17* [1.31–5.72]	0.005	2.67* [1.23–5.14]	<0.001	2.54* [1.19–4.87]	0.001	3.37* [1.41–8.03]	<0.001	3.62* [1.88–5.87]	<0.001	3.02* [1.32–6.81]	0.007
Grocery shopping	1.25 [0.88–1.53]	0.056	1.29 [0.96–1.74]	0.084	1.08 [0.81–1.43]	0.616	1.16 [0.71–1.97]	0.726	1.14 [0.72–1.97]	0.381	1.21 [0.82–2.17]	0.186
Self-assessed diet quality	3.92* [1.54–6.46]	<0.001	3.33* [1.78–7.16]	<0.001	3.32* [1.96–4.96]	<0.001	2.18* [1.26–4.92]	0.008	2.36* [1.47–5.84]	<0.001	2.76* [1.35–6.39]	0.000
Nutrition knowledge	2.36* [1.28–5.78]	0.001	2.29* [1.22–4.13]	0.009	2.17* [1.14–5.51]	0.008	3.28* [2.21–4.96]	<0.001	1.69* [1.27–2.22]	0.021	1.63* [1.22–2.19]	0.001
Label’s awareness	1.94* [1.25–4.30]	0.012	1.78* [1.08–3.26]	0.034	1.84* [1.16–4.25]	0.023	2.02* [1.12–4.68]	0.009	2.26* [1.23–4.36]	<0.001	1.92* [1.17–3.54]	0.008

CWO, Chilean Warning Octagons; HSR, Health Star Rating system; MTL, Multiple Traffic Lights; NS, Nutri-Score; OR: Odds Ratio; CI: Confidence Interval. * Significant at 5% level of significance.

Furthermore, the results from the ordinal logistic models demonstrated that nutritional food choices were significantly and positively associated with education, income, self-assessed diet quality, self-assessed nutrition knowledge, and nutrition awareness at the time of survey completion across all food categories and within each food category. The estimation results also revealed statistically significant positive associations between age and nutritional food choices for the overall model (OR = 1.95, 95% C.I: [1.41–2.86], *p*-value = 0.008), cheese (OR = 1.74, 95% C.I: [1.29–2.33], p-value = 0.008), cereals (OR = 1.46, 95% C.I: [1.11–2.86], *p*-value = 0.019), and bread (OR = 1.59, 95% C.I: [1.19–2.13], *p*-value = 0.016) models. However, no significant associations were observed between nutritional food choices and gender, marital status, or responsibility for grocery shopping.

### 3.4 Results of objective understanding analysis

**Figure 4:**
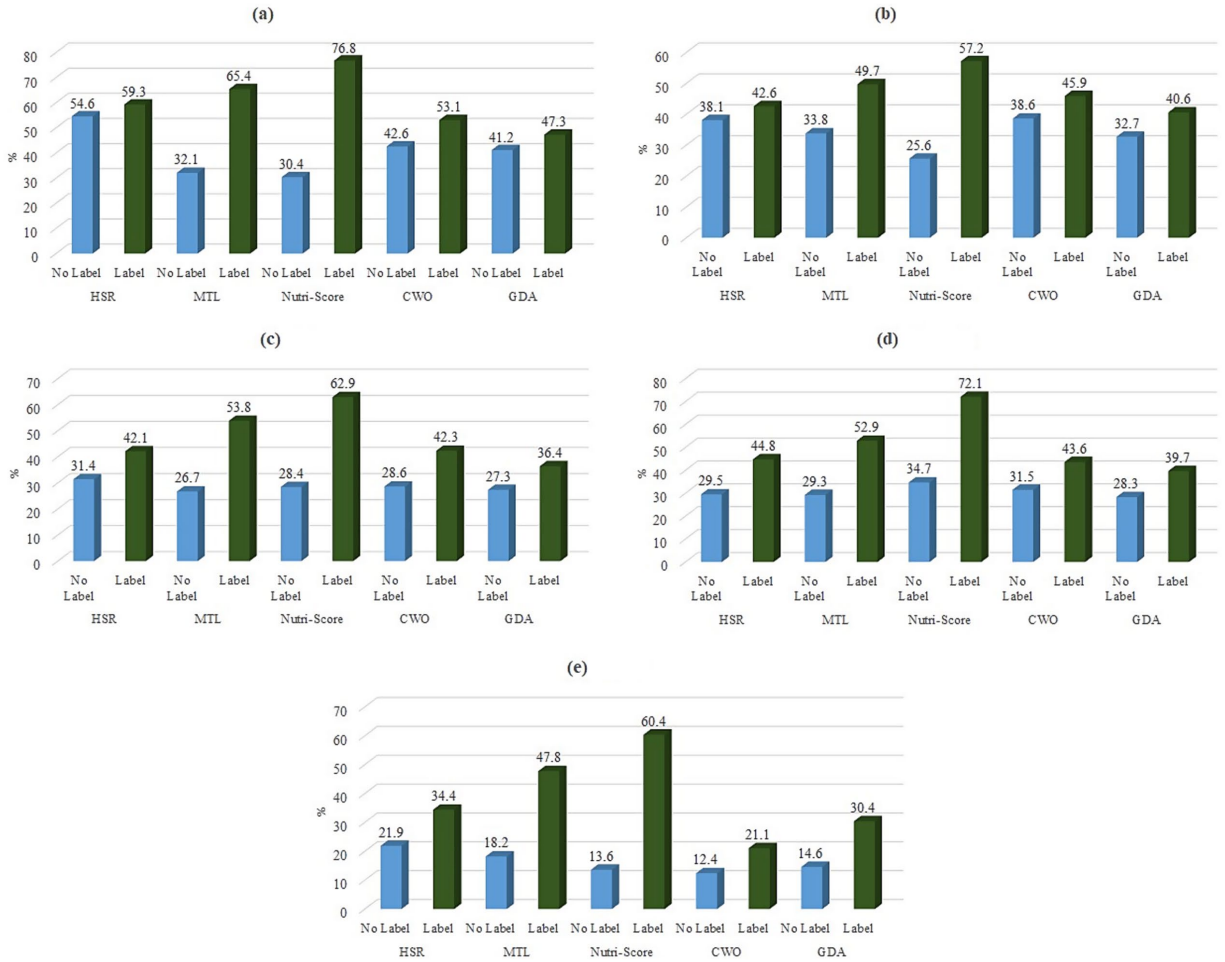
Percentage of correct answers for ranking tasks, categorized by FoPL type and food category. (a): Cheese. (b): Nuggets. (c): Juice. (d): Cereals. (e): Bread.

Figure 4 illustrates the percentages of correct answers in both the no-label and label conditions, classified according to FoPL type and food category. The results revealed that compared to the no-label condition, all FoPLs improved the percentage of correct answers, though there were some differences between the label formats. Across all five food categories, the NS showed the most substantial improvement in correct answers in the ranking tasks, followed by the MTL. The performance of the other FoPLs varied depending on the particular food category.

The correlation coefficients between participants’ sociodemographic and behavioral characteristics and understanding scores are displayed in Supplementary Table S2. The analyses revealed that NS consistently exhibited higher positive correlations across the product types, followed by MTL and CWO with the understanding score. The findings also revealed that the score of objective understanding positively correlated with education level, self-reported diet quality, nutrition knowledge, and label awareness at the time of the survey, across all food categories. This suggests that these characteristics can help improve consumers’ understanding of FoPLs. However, age, sex, marital status, household monthly income level, and responsibility for grocery shopping were not significantly correlated with the understanding score.

Table 4 illustrates the associations between respondents’ ability to correctly rank goods by nutritional quality and FoPL, along with demographic and behavioral factors, based on the estimation results from the multivariate ordinal logistic regression analyses. All models showed strong performance and successfully met all diagnostic criteria. The results revealed that the NS exhibited the most significant improvement in participants’ ability to correctly rank products according to their nutritional quality compared to the GDAs (OR = 5.81, 95% CI: 2.92 to 7.28, *p*-value < 0.001) across all food categories. It was followed by the MTL (OR = 3.16, 95% CI: 1.58 to 5.81, *p*-value < 0.001) and the CWO (OR = 2.08, 95% CI: 1.35 to 2.39, *p*-value = 0.027). When the analyses were conducted by food category, the NS exhibited superior performance across all five food types and particularly emerged as the only FoPL to demonstrate significant improvements compared to the GDAs label for nuggets (OR = 3.19, 95% CI: 2.14 to 5.13, *p*-value < 0.001), cheese (OR = 2.73, 95% CI: 1.86 to 4.47, *p*-value < 0.001), and cereals (OR = 2.56, 95% CI: 1.79 to 3.47, *p*-value < 0.001). In the juice category, the NS performed the best (OR = 4.76, 95% CI: 2.85 to 6.58, *p*-value < 0.001) compared to the GDAs label, followed by the MTL (OR = 2.41, 95% CI: 1.54 to 4.15, *p*-value < 0.001) and CWO (OR = 1.53, 95% CI: 1.08 to 2.24, *p*-value = 0.032) in terms of performance. For the bread category, participants scored the highest with the NS (OR = 2.83, 95% CI: 2.08 to 4.39, *p*-value < 0.001), followed by MTL (OR = 2.46, 95% CI: 1.55 to 4.28, *p*-value < 0.001) compared to the GDA’s label.

**Table 4:** Multivariate ordinal logistic regression results for the ability to correctly rank products according to nutritional quality across food categories.

Variables	All food types		Cheese		Nuggets		Juice		Cereals		Bread	
	OR [95% C.I]	*p*-value	OR [95% C.I]	*p*-value	OR [95% C.I]	*p*-value	OR [95% C.I	*p*-value	OR [95% C.I]	*p*-value	OR [95% C.I]	*p*-value
Type of FoPL												
HSR	1.32 [0.93–2.16]	0.074	1.54 [0.71–2.04]	0.142	1.39 [0.77–2.63]	0.142	1.46 [0.59–2.64]	0.135	1.19 [0.79–1.78]	0.718	1.54 [0.79–3.12]	0.317
MTL	3.16* [1.58–5.81]	<0.001	1.43 [0.91–2.51]	0.079	1.41 [0.87–2.49]	0.117	2.41* [1.54–4.15]	<0.001	1.34 [0.91–2.08]	0.315	2.46* [1.55–4.28]	<0.001
NS	5.81* [2.92–7.28]	<0.001	2.73* [1.86–4.47]	<0.001	3.19* [2.14–5.13]	<0.001	4.76* [2.85–6.58]	<0.001	2.56* [1.79–3.74]	<0.001	2.83* [2.08–4.39]	<0.001
CWO	2.08* [1.35–2.39]	0.027	1.23 [0.85–2.14]	0.431	1.31 [0.78–2.18]	0.253	1.53* [1.08–2.24]	0.032	1.13 [0.75–1.71]	0.893	1.05 [0.67–1.65]	0.926
Male	0.87 [0.41–1.79]	0.492	1.29 [0.72–2.63]	0.526	1.38 [0.72–2.73]	0.486	1.09 [0.26–2.48]	0.829	0.94 [0.42–1.36]	0.762	1.14 [0.46–2.15]	0.428
Marital status												
Married	1.34 [0.91–2.16]	0.152	1.28 [0.88–1.94]	0.174	1.21 [0.77–1.96]	0.309	1.32 [0.81–2.08]	0.242	1.21 [0.81–1.82]	0.341	1.25 [0.82–1.89]	0.347
Divorced	0.95 [0.62–1.58]	0.847	0.91 [0.57–1.43]	0.643	0.88 [0.54–1.39]	0.552	0.93 [0.61–1.61]	0.788	0.89 [0.55–1.62]	0.738	0.91 [0.54–1.67]	0.779
Widowed	1.14 [0.75–1.79]	0.561	1.06 [0.71–1.79]	0.509	1.09 [0.66–1.78]	0.691	1.11 [0.64–1.91]	0.687	1.07 [0.61–1.88]	0.832	1.08 [0.59–1.81]	0.766
Age	1.07 [0.88–1.36]	0.223	1.11 [0.91–1.41]	0.195	1.14 [0.93–1.46]	0.174	1.05 [0.82–1.31]	0.321	0.96 [0.79–1.32]	0.571	1.18 [0.46–2.15]	0.481
Education	3.59* [1.62–6.98]	<0.001	2.44* [1.12–5.14]	0.021	3.01* [1.59–5.51]	0.007	2.68* [1.35–5.16]	0.021	2.12* [1.09–4.04]	0.017	2.89* [1.56–5.18]	0.003
Income level	1.18 [0.92–1.61]	0.217	1.23 [0.87–1.74]	0.245	1.14 [0.79–1.62]	0.446	1.32 [0.87–1.98]	0.227	1.16 [0.81–1.48]	0.453	1.11 [0.85–1.47]	0.327
Grocery shopping	1.15 [0.76–1.72]	0.513	1.15 [0.76–1.72]	0.513	1.08 [0.60–1.88]	0.818	1.10 [0.68–1.76]	0.645	1.32 [0.77–2.28]	0.300	1.16 [0.77–1.83]	0.684
Self-assessed diet quality	4.60* [2.21–7.86]	<0.001	5.42* [2.23–12.95]	<0.001	4.50* [2.04–9.52]	<0.001	3.90* [1.80–8.49]	<0.001	4.02* [2.06–7.85]	<0.001	5.71* [2.62–12.45]	<0.001
Nutrition knowledge	2.58* [1.36–5.89]	<0.001	2.31* [1.21–4.84]	0.005	2.15* [1.04–4.49]	0.037	1.91* [1.01–4.21]	0.042	2.46* [1.48–6.27]	<0.001	2.11* [1.16–3.98]	0.015
Label’s awareness	2.94* [1.33–5.72]	0.003	2.48* [1.21–4.91]	0.004	2.38* [1.16–4.89]	0.008	2.26* [1.12–4.47]	0.015	2.13* [1.18–4.89]	0.006	2.46* [1.32–4.82]	0.004

CWO, Chilean Warning Octagons; HSR, Health Star Rating system; MTL, Multiple Traffic Lights; NS, Nutri-Score; OR, Odds Ratio; CI, Confidence Interval. * Significant at 5% level of significance.

The estimation results further indicated that education, self-assessed nutritional quality, self-assessed knowledge, and awareness of the label during survey completion were significant factors enhancing respondents’ ability to accurately rank food by nutritional quality, across the overall sample and within each food category. However, the ability to accurately rank food choices was not significantly associated with age, gender, income, marital status, or responsibility for grocery shopping.

## 4 Discussion

The current study examined how Saudi consumers perceive and understand five distinct FoPLs, which include the HSR, MTL, NS, GDA, and CWO, and how these labels influence their food choices. Generally, among the five different FoPLs examined in the study, the current analyses revealed that the NS emerged as the most effective system in encouraging healthier food choices among respondents and enhancing their ability to differentiate nutritional quality differences within specific product categories. This result is congruent with several previous studies conducted in Switzerland (37) and France (46, 47), where experimental investigations involving consumers in shopping tasks, with or without a FoPL, demonstrated that the NS proved to be the most effective in enhancing the nutritional quality of purchases compared to various systems such as MTLs and warning symbols (37, 46, 47). Our findings also confirmed several internationally comparative studies that demonstrated the importance of the NS model in improving consumers’ awareness and buying choices (18, 36, 38). However, the findings from previous studies conducted in Canada and Uruguay indicated that warning symbols were more effective in improving the nutritional quality of product purchases among consumers from these two countries, which is not consistent with our findings (48, 49). Meanwhile, this study revealed discrepancies in food choices across different FoPL models for cereals and nuggets. These variations in effectiveness may be attributed to consumer perceptions, label design, cognitive effort, cultural influences, and health risk awareness. Familiar foods like cereals align better with numerical labels (GDA), whereas processed foods (e.g., nuggets) benefit more from color-coded warnings (MTL). Implementing tailored labeling strategies can enhance effectiveness across diverse food categories (50).

Several previously published studies have investigated the influence of different FoPLs on the nutritional quality of consumers’ food choices or purchases. These studies have produced varied results, which can be linked to the particular FoPLs under scrutiny and the research methodologies utilized such that these studies highlighted that FoPLs can have a modest yet notable positive impact on the nutritional quality of food choices and purchases (17, 24, 29, 36, 44, 51–59). Previous research also pointed out that labeling systems, including but not limited to NS (38, 39, 47, 53), MTLs (29, 53, 57, 60), HSR (37, 58), and warning symbols (29, 44, 49, 55, 59) seem to be linked with more health-conscious food choices. For example, Egnell et al. (44) found that no significant association was observed in terms of changes in the nutritional quality of food selections, both overall and within particular food categories as compared to the reference intake labels among Dutch participants, but the warning label stood out as an exception to this pattern, as it promoted consumers to choose a healthier breakfast cereal (44). Furthermore, the findings of comparative research that examined the proportional impacts of different label types suggested that there are only minor variations between various FoPLs in terms of their influence on food choices (37, 46, 47). Nonetheless, it is crucial to acknowledge that prior research did not conduct experimental investigations or comparative analyses involving real or actual products to evaluate the influence of FoPLs on consumer purchases. Consequently, it is prudent to approach the interpretation of the findings with caution. Egnell et al. (37) suggested that when comparative experimental studies evaluating the impact of FoPLs on food purchases in physical or online shops are not accessible, the most effective approach would be to focus on comparing how different FoPLs assist consumers in comprehending the nutritional quality of foods.

The current study highlighted the significance of socio-economic and behavioral characteristics on food choices. Participants with higher education and income levels, rated their diet quality as healthy, nutrition knowledge, or aware about the nutrition label were more likely to improve their food choice scores across and within all food categories, reflecting their better access to information and resources relating to health, as well as being better able to make choices based on that information. Findings from Egnell et al. (37) supported our results by demonstrating that food category and income were significantly associated with food choice scores, not only across all food categories but also specifically for products such as pizzas, cakes, and breakfast cereal. The study also showed that older respondents were likely to have made healthier food choices for the entire sample, as well as for some food categories like bread, cheese, and cereals. This pattern may reflect a greater emphasis on health among older individuals or the development of more consistent healthy eating habits over time. A study by AlbuObaid and Al-Mahish (45) revealed that increased age and awareness among Saudi consumers were among the significant contributing factors to their decision to buy products with FoPL. Meanwhile, Szakos et al. (61) suggested that most older adults engage with nutrition primarily in response to existing health conditions, rather than as a proactive or preventive approach to sustaining long-term health. These findings highlight the importance of promoting nutrition education and FoPL, particularly among lower-income and younger populations, to encourage healthier food choices.

Furthermore, the findings of this study about objective understanding enabled differentiation among various FoPLs, with the NS demonstrating superior performance compared to other labeling systems, followed by MTL as compared to GDA label. Our results are congruent with several global studies that employed the same methods and demonstrated that NS possessed a considerably higher ability in assisting participants to correctly rank the overall nutritional quality of food items (18, 29, 36, 37, 44, 60, 62–65). Previous research suggested that summary indicators of food nutritional labels are more consumer-friendly (65–67), while nutritional mechanisms that rely largely on numerical quantitative labels specific to individual nutrients demand a higher cognitive effort from consumers that can impede their understanding and use in purchasing items (37, 44). Using color coding, particularly the red, yellow, and green spectrum, offers an easily comprehensible signal for “stop” and “go” indications (66). Studies have also shown that employing this method improves the ability to capture attention (62, 67). Therefore, various previous studies suggested that a FoPL integrating both summarized information and color-coded components, like the NS, is linked with enhanced and improved objective comprehension among consumers (37, 39, 44, 65, 68). The alignment of the NS effects on food choices and objective understanding of their ability to correctly rank food products in this study suggested that it may prove to be an effective and valuable intervention for Saudi consumers.

The analysis also suggested that education, nutrition knowledge, diet quality, and label awareness significantly enhance respondents’ ability to accurately rank food products by nutritional value, with effects varying across food categories. These findings suggest that improving nutrition education and awareness of FoPLs can enhance consumers’ ability to make healthier food choices (37, 69, 70). Egnell et al. (37) highlighted that healthier self-assessed diet quality and younger age significantly contributed to an improved ability to accurately rank products among the Swiss cohort, with the magnitude of the effect varying across specific food categories. Grunert et al. (70) suggested that nutrition knowledge is the main contributing factor to understanding the nutrition information on food labels.

The current study also showed that the majority of participants stated that they had knowledge about nutrition, food, and beverages, and were aware of the importance of food labels. This study also found that less than half of the participants reported occasionally reading food labels when purchasing a product to be aware of harmful or sensitive ingredients. The findings of this study align with various previously reported studies (29, 37, 44). For example, the seminal work of Egell et al. showed that 28% of Swiss consumers, 16% of Dutch consumers, and 20.8% of German consumers had little or no nutritional knowledge (37, 44). Furthermore, approximately one-quarter of the participants were unable to recall having seen food labels, with the highest proportion of such instances occurring among those assigned to the GDA label group. Similar results were also observed regarding the recollection of having seen food labels during the survey among consumers from Dutch, German, and Swiss backgrounds. However, the highest percentage among German and Dutch participants was found in the warning symbol scheme (37, 44), while for Swiss consumers, the HSR scheme had the highest percentage (18).

In the current study, 21.6% of participants reported a lack of nutrition knowledge, while 13.8% stated that they were unfamiliar with food and beverage labels. This aligns with previous findings from a cross-sectional survey conducted across four Arab countries, where 72.8% of participants in Saudi Arabia exhibited unsatisfactory levels of nutritional knowledge. The study further reported that female gender, education, and reading nutrition articles were significantly associated with adequate knowledge (*p* < 0.001) (71). To bridge these gaps, policymakers could implement targeted educational campaigns aimed at improving the understanding of nutrition labels. Moreover, school nutrition education can ensure better nutritional literacy.

In this study, 11.1% of participants cited the availability of food labels in English only as a reason for not reading them. A similar language barrier regarding food labels was noted in a previous study conducted in Saudi Arabia (40). It is important to contextualize our findings within the Saudi Arabian context, as food labels in English may limit access to information for specific groups of the population, particularly individuals with lower literacy levels or the older adult who are less familiar with English.

The main strength of this study is notably attributed to the utilization of a randomized design to recruit participants, allowing for a thorough assessment of how various FoPL designs compare across their food choices and objective understanding. This approach enhances the reliability of our findings in multiple ways. First, it eliminates the selection bias, ensuring that each FoPL type is allocated to participants without any influence from pre-existing characteristics or preferences and thus improves the internal validity of the current findings. Second, it helped us to thoroughly compare the influence of different FoPLs on consumers’ food choices and objective understanding, offering a comprehensive understanding of how these diverse designs influence consumer behavior and thus making our research valuable for both policymakers and stakeholders. Another important strength is the large and diverse sample of Saudi consumers, which increases the power and robustness of the statistical analyses conducted.

Nevertheless, the current study has some potential limitations. Firstly, we employed quota sampling and online survey methods to recruit participants, which may limit the generalizability of our findings to a broader population. However, it’s worth noting that by using quota sampling, we ensured equitable representation of the sample across various socio-economic groups. Second, since the participants were unaware of the study hypotheses, they were not given any information regarding the purpose or significance of the FoPL they were exposed. This lack of information could potentially lead to an underestimation of the impact of FoPL labels. Third, participants were not exposed to real-life food products, which might affect their performance in correctly ranking food in the objective understanding task. Fourth, this study did not take into account participants’ perceptions of FoPLs and therefore future studies are encouraged to address this limitation to improve the ecological validity of the consumers’ perceptions about different types of FoPLs.

We acknowledge that recruiting participants only from public places in Riyadh may limit the generalizability of our findings to the broader Saudi Arabian population, particularly in rural areas and other regions. Future studies should include participants from rural areas to enhance the diversity and representativeness of the sample. Additionally, we recommend using real food products to improve ecological validity and better reflect actual consumer behavior. Moreover, use of objective measures of knowledge and food choices may be considered to complement self-reported data and reduce bias. The current study employed a rigorous methodology to draw meaningful conclusions regarding the effects of various types of FoPLs, offering valuable insights to advance the field of research in nutrition labeling and consumer behavior. Nevertheless, caution should be exercised in generalizing the experimental results to actual consumer behavior in real-life settings.

## 5 Conclusion

Our results demonstrated that NS exhibited the most significant enhancement in food choice improvements and proved to be the most effective in helping consumers rank products according to their overall nutritional quality, thereby empowering them to make healthier food choices. This finding has important implications for policymakers in Saudi Arabia, particularly in light of the high obesity rates in the country. The SFDA may consider incorporating NS into its nutritional labeling policies. Additionally, future policies should consider not only the adoption of NS but also nutritional education programs to increase consumer engagement with food labeling systems. With its simple, color-coded system, NS can help overcome barriers to healthy eating, such as the lack of consumer understanding of complex nutritional information on food labels and the time spent on interpreting them.

## Data Availability

The raw data supporting the conclusions of this article will be made available by the authors, without undue reservation.

## Footnotes

^1^https://www.qub.ac.uk/elearning/public/HealthyEating/FoodLabellingandGDAs/

^2^ https://www.qub.ac.uk/elearning/public/HealthyEating/FoodLabellingandGDAs/

^3^ https://healthpolicy-watch.news/chile-provides-a-convincing-case-for-mandatory-warning-labels-on-processed-food/

^4^ https://www.santepubliquefrance.fr/en/nutri-score

^5^ https://www.healthstarrating.gov.au/
