# Supplementary material for: Comparing the influence of front-of-pack nutrition labels on Saudi consumers’ understanding and food selection
Source: Front Public Health. 2025 Jun 3;13:1527531. doi: 10.3389/fpubh.2025.1527531 (PMC12171830; doi:10.3389/fpubh.2025.1527531)
Supplement: Supplementary file 1 [file Data_Sheet_1.docx]

**Table S1.** Spearman’s correlation coefficients between participants’ characteristics and food choice scores across food categories

| **Variables** | **All food types** | **Cheese** | **Nuggets** | **Juice** | **Cereals** | **Bread** |
| --- | --- | --- | --- | --- | --- | --- |
| Type of FoPL |  |  |  |  |  |  |
| HSR | 0.31* | 0.29* | 0.36 | 0.21* | 0.07 | 0.26* |
| MTL | 0.12 | 0.18* | 0.28* | 0.11 | 0.04 | 0.10 |
| NS | 0.39* | 0.33* | 0.23* | 0.33* | 0.37* | 0.03 |
| CWO | 0.11 | 0.14 | 0.10 | 0.06 | 0.11 | 0.34* |
| GDA | 0.08 | 0.09 | 0.04 | 0.02 | 0.10 | 0.06 |
| Sex | - 0.08 | - 0.04 | 0.03 | 0.02 | 0.43* | 0.00 |
| Marital status |  |  |  |  |  |  |
| Married | 0.05 | 0.07 | - 0.11 | 0.11 | 0.06 | 0.07 |
| Divorced | - 0.08 | - 0.03 | - 0.08 | - 0.04 | 0.02 | 0.06 |
| Widowed | - 0.03 | 0.08 | 0.04 | 0.02 | 0.09 | 0.02 |
| Age | 0.22* | 0.26* | 0.06 | 0.08 | 0.26* | 0.31* |
| Education | 0.36* | 0.27* | 0.25* | 0.32* | 0.29* | 0.35* |
| Income level | 0.42* | 0.41* | 0.28* | 0.38* | 0.31* | 0.29* |
| Grocery shopping | 0.09 | 0.11 | 0.06 | 0.09 | 0.05 | 0.10 |
| Self-assessed diet quality | 0.41* | 0.31* | 0.36* | 0.33* | 0.27* | 0.34* |
| Nutrition knowledge | 0.34* | 0.38* | 0.43* | 0.36* | 0.39* | 0.41* |
| Label’s awareness | 0.35* | 0.32* | 0.29* | 0.31* | 0.38* | 0.32* |
| CWO: Chilean Warning Octagons; GDA: Guideline Daily Amount; HSR: Health Star Rating system; MTL: Multiple Traffic Lights; NS: Nutri-Score; OR: Odds Ratio; CI: Confidence Interval.  * Significant at 5% level of significance. | | | | | | |

**Table S2.** Spearman’s correlation coefficients between participants’ characteristics and the ability to correctly rank products according to nutritional quality across food categories

| **Variables** | **All food types** | **Cheese** | **Nuggets** | **Juice** | **Cereals** | **Bread** |
| --- | --- | --- | --- | --- | --- | --- |
| Type of FoPL |  |  |  |  |  |  |
| HSR | 0.28* | 0.21 | 0.20 | 0.23 | 0.21 | 0.24 |
| MTL | 0.35* | 0.20 | 0.19 | 0.33* | 0.20 | 0.34* |
| NS | 0.43* | 0.34* | 0.36* | 0.40* | 0.32* | 0.34* |
| CWO | 0.30* | 0.19 | 0.17 | 0.25* | 0.17 | 0.15 |
| GDA | 0.22* | 0.08 | 0.05 | 0.12 | 0.34* | 0.31* |
| Sex | - 0.08 | 0.06 | 0.10 | 0.03 | - 0.07 | 0.04 |
| Marital status |  |  |  |  |  |  |
| Married | 0.11 | 0.09 | 0.10 | 0.08 | 0.06 | 0.09 |
| Divorced | - 0.05 | - 0.07 | - 0.04 | - 0.05 | - 0.01 | - 0.02 |
| Widowed | 0.07 | 0.03 | 0.05 | 0.02 | 0.08 | 0.06 |
| Age | 0.12 | 0.08 | 0.12 | 0.11 | 0.09 | 0.10 |
| Education | 0.43* | 0.33* | 0.37* | 0.34* | 0.31* | 0.36* |
| Income level | 0.09 | 0.03 | 0.11 | 0.04 | 0.00 | 0.08 |
| Grocery shopping | 0.11 | 0.10 | 0.09 | 0.08 | 0.03 | 0.07 |
| Self-assessed diet quality | 0.42* | 0.44* | 0.41* | 0.39* | 0.40* | 0.45* |
| Nutrition knowledge | 0.31* | 0.29* | 0.27* | 0.25* | 0.31* | 0.27* |
| Label’s awareness | 0.37* | 0.31* | 0.29* | 0.28* | 0.27* | 0.31* |
| CWO: Chilean Warning Octagons; GDA: Guideline Daily Amount; HSR: Health Star Rating system; MTL: Multiple Traffic Lights; NS: Nutri-Score.  * Significant at 5% level of significance. | | | | | | |
